# Reader bias in breast cancer screening related to cancer prevalence and artificial intelligence decision support—a reader study

**DOI:** 10.1007/s00330-023-10514-5

**Published:** 2024-01-02

**Authors:** Hanen Al-Bazzaz, Marina Janicijevic, Fredrik Strand

**Affiliations:** 1 Mälarsjukhuset Eskilstuna, Kungsvägen 42, 633 49, Eskilstuna, Sweden; 2 Department of Oncology-Pathology, Karolinska Institutet, L2:03, Karolinska Vägen 8, 171 64 Solna, Sweden; 3 Breast Radiology, Medical Diagnostics Karolinska, Karolinska University Hospital, NB1:03, Gävlegatan 55, 171 76 Stockholm, Sweden

**Keywords:** Breast, Cancer screening, Mammography, Artificial intelligence, Bias

## Abstract

**Objectives:**

The aim of our study was to examine how breast radiologists would be affected by high cancer prevalence and the use of artificial intelligence (AI) for decision support.

**Materials and method:**

This reader study was based on selection of screening mammograms, including the original radiologist assessment, acquired in 2010 to 2013 at the Karolinska University Hospital, with a ratio of 1:1 cancer versus healthy based on a 2-year follow-up. A commercial AI system generated an exam-level positive or negative read, and image markers. Double-reading and consensus discussions were first performed without AI and later with AI, with a 6-week wash-out period in between. The chi-squared test was used to test for differences in contingency tables.

**Results:**

Mammograms of 758 women were included, half with cancer and half healthy. 52% were 40–55 years; 48% were 56–75 years. In the original non-enriched screening setting, the sensitivity was 61% (232/379) at specificity 98% (323/379). In the reader study, the sensitivity without and with AI was 81% (307/379) and 75% (284/379) respectively (*p* < 0.001). The specificity without and with AI was 67% (255/379) and 86% (326/379) respectively (*p* < 0.001). The tendency to change assessment from positive to negative based on erroneous AI information differed between readers and was affected by type and number of image signs of malignancy.

**Conclusion:**

Breast radiologists reading a list with high cancer prevalence performed at considerably higher sensitivity and lower specificity than the original screen-readers. Adding AI information, calibrated to a screening setting, decreased sensitivity and increased specificity.

**Clinical relevance statement:**

Radiologist screening mammography assessments will be biased towards higher sensitivity and lower specificity by high-risk triaging and nudged towards the sensitivity and specificity setting of AI reads. After AI implementation in clinical practice, there is reason to carefully follow screening metrics to ensure the impact is desired.

**Key Points:**

*• Breast radiologists’ sensitivity and specificity will be affected by changes brought by artificial intelligence.*

*• Reading in a high cancer prevalence setting markedly increased sensitivity and decreased specificity.*

*• Reviewing the binary reads by AI, negative or positive, biased screening radiologists towards the sensitivity and specificity of the AI system.*

**Graphical abstract:**

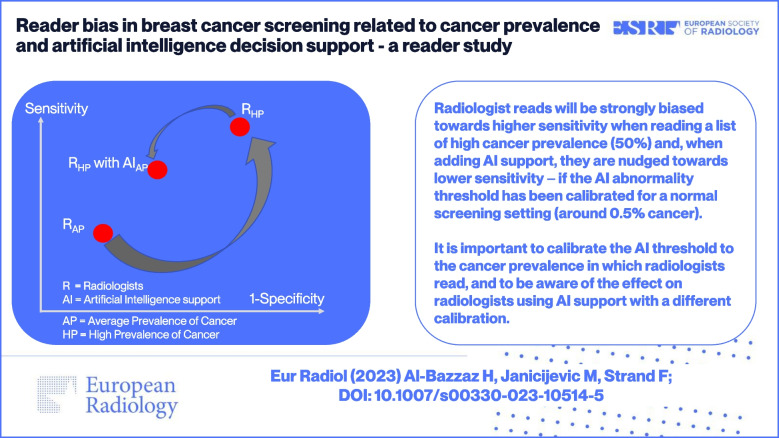

## Introduction

Artificial intelligence (AI) for computer-aided detection (CAD) is increasingly seen as a realistic option for alleviating radiologist shortage and for increasing cancer detection [1, 2]. Retrospective studies have shown cancer detection on par with breast radiologists and reader studies have demonstrated improved reader performance with AI support [3].

There are at least three alternative implementations of AI CAD. First, AI can be implemented as an independent reader, replacing one of two radiologists in double-reading [4]. Second, AI can be implemented as a triage tool, assigning each exam to a category related to expected cancer prevalence [5, 6]. Third, it can be implemented as a concurrent assistant providing decision support to the radiologist before they make their decision to flag an abnormality [7]. The second option may cause a subconscious change in the operating point of the radiologist due to cancer enrichment, i.e., an increased proportion of cancer in the reading list compared to the regular population-average reading that benchmarks are based on. The third option implies the most interaction with the radiologist and warrants adequate reader studies before clinical implementation to understand how different readers are affected and how the tendency to erroneously change their assessment is influenced by the type of AI information provided and by what image signs they have observed themselves. Recently, there have been reports of severe automation bias when radiologists tend to follow the erroneous suggestions of AI in reading screening mammograms [8, 9]. The tendency to follow an erroneous AI suggestion was present for both positive and negative exams, and was more pronounced for less experienced radiologists. Understanding how interpreting radiologists are affected by AI information is increasingly important, as AI has potential to be involved not only in cancer detection but also to extract image biomarkers for density assessment, radiological-pathological correlation, and predicting therapy response [10].

To further increase understanding of how a cancer-enriched setting and AI decision support may influence radiologists, we conducted a reader study using a reading list with a large proportion of cancer and performed the full double-reading and consensus discussion first without AI decision support and later with AI decision support.

## Methods

### Study setting

The ethical review board had approved the study and had waived the need for individual informed consent. This retrospective reader study was based on the publicly available cancer-enriched case–control population CSAW-CC (10.5878/45vm-t798) [11] from which we extracted the most recent mammography exam before diagnosis for 379 randomly selected women diagnosed with breast cancer and 379 randomly selected healthy women. Screening mammograms had been acquired between 2010 and 2013 from Karolinska University Hospital, Sweden. The diagnostic reference standard for each exam, healthy or breast cancer, was defined by linking to the Stockholm-Gotland regional cancer center breast cancer registry where breast cancer diagnoses are based on pathology verification. We considered a diagnosis date within a 2-year follow-up period after screening as the reference standard of having breast cancer. Age was only available as two categories: 40 to 55 years of age (“younger”) and 56 to 75 years of age (“older”). Karolinska invites all women between 40 and 74 years of age to mammography screening every 2 years based on having a home address in the catchment area of the hospital.

### Images

For each screening exam, two standard views, craniocaudal and mediolateral oblique, had been acquired for each breast on full-field digital mammography Hologic equipment.

### Original screening reads

For the historic screening reads, we extracted from the hospital radiology system the reads by the original two screening radiologists and the recall decision from the consensus discussion for each exam. If any of the two is flagged for abnormality, the exam would go to a consensus discussion where two radiologists together decide whether the woman should be recalled for further work-up or sent a “healthy letter.” The original screening radiologists had access to images, biopsy history, and any symptom reported by the woman at the point of screening. In Sweden, even women reporting a symptom to the radiographer at the time of imaging remain part of the screening population and are not considered a clinical patient.

### AI system

The AI system used for the reader study was Insight MMG (Lunit Inc.). In preparation for the reader study, for all mammography images, the AI system generated image prompts linked to abnormality scores between 0 and 100. The highest AI score in any of the four standard views defined the exam-level AI score. For a binary classification into flag or no-flag, we used a pre-calibrated threshold value of 40 which had been defined in a prior study to match the specificity of the average screening radiologist in regular, non-enriched, population-wide screening [4]. In the reader study session with AI, the readers and consensus discussion had access to all of the above information when making their own assessments.

### Reader study protocol

**Fig. 1:**
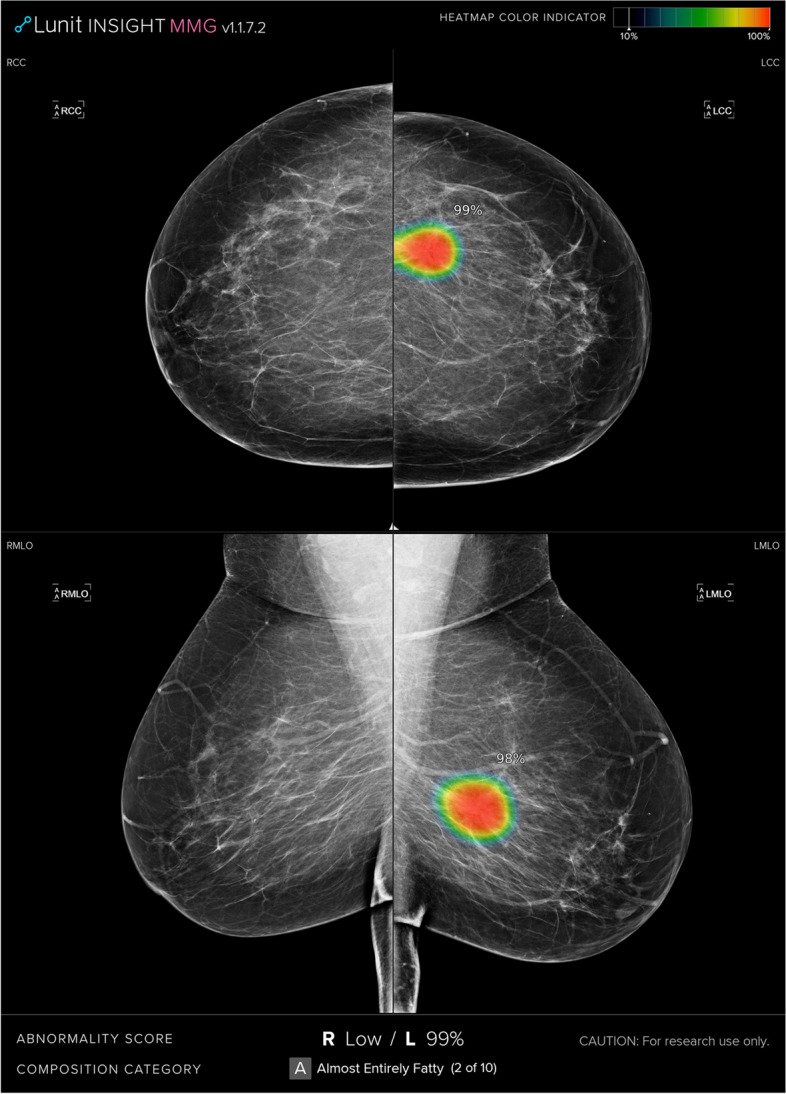
Example of how the AI information was visualized for the radiologists in the reader study. For all exams, the highest AI score of the right and left breast was shown at the bottom of the four images (in this example 99% for the left breast). In addition, if the AI score was 10% or higher, the suspicious location was shown by a prompt in the image (in this example mainly in red color)

Exams were reviewed in two sessions—without AI and with concurrent AI decision support—separated by a 6-week wash-out period. Images were shown on an Eizo Radiforce RX560 monitor. Study radiologists, as well as AI, did not have access to prior images, prior biopsy history, and other patient information and were not informed about the proportion of cancer in the population. For the session with AI, the radiologists had access to the AI score on exam-level and by AI image prompts showing the location and score of individual image findings (Fig. 1). For both sessions, without and with AI, two breast radiologists (M.J. and another radiologist; each reading half of the exams) handled the first read, one breast radiologist handled the second read (H.A.), and consensus discussions were performed by two out of these three radiologists. The experience level counted as breast imaging–specific work time was more than 5 years for HA and more than 10 years for the other two radiologists. The second read was blinded in relation to the first read. An exam that received a flag of an abnormality in any of the two reads was further assessed in a discussion between two of the three study radiologists. In addition to flagging for abnormality, the radiologists ranked the exam on a scale of 1 to 5, similar to Bi-RADS categories, where 1 indicated that no lesion was present and 5 indicated that a lesion was perceived as “definitely malignant.” Any suspicious lesion was categorized according to mammographic signs: mass, microcalcifications, architectural distortion, asymmetrical density, nipple retraction, skin thickening, suspicious axillary lymph node. For each read, the reader measured the time in seconds by using a stop-watch which was started when images were displayed and stopped when a flag or no-flag decision was made, not including the time for further characterizing the image findings and writing the full assessment.

We analyzed three different read-and-consensus regimens: the original screening radiologists, the study radiologists not using AI, and the study radiologists using concurrent AI decision support in terms of binary positive and negative reads of each exam accompanied by image markers. For each regimen, we calculated flagging rate (exams flagged by any reader divided by all exams) and recall rate (exams with recall decision divided by all exams), and then, for both flagged exams and recalled women, we calculated positive predictive value (proportion of positive exams where cancer was diagnosed), sensitivity (proportion of cancer diagnosed with a positive read), specificity (proportion of healthy exams without a positive reads), and radiologist workload. In addition, we examined occurrences of recall decisions that changed with the use of AI in relation to the original Bi-RADS category and the mammographic signs of suspected lesions.

### Statistical analysis

Stata version 15.1 (StataCorp) was used for all statistical tests and estimations. The chi-squared test was used to test for differences in contingency tables. All statistical tests were two-sided. The level for statistical significance was set at *α* = 0.05, which was not adjusted for multiple comparisons.

## Results

### Study population

**Fig. 2:**
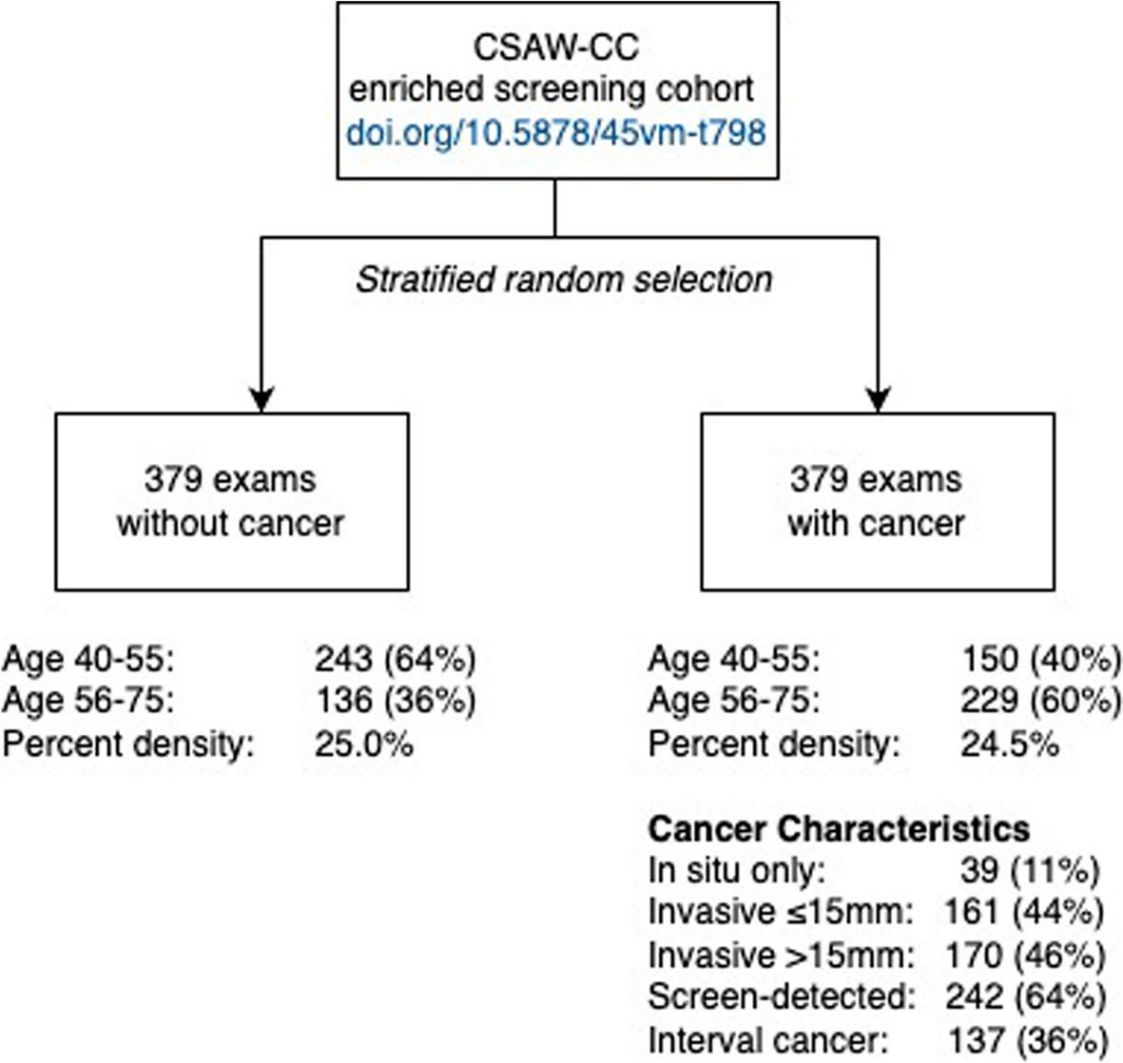
Exam selection flowchart. Using the anonymized CSAW-CC screening cohort, we randomly selected 379 women with cancer diagnosed within 2 years of the mammogram and 379 women who remained healthy 2 years after the mammogram

We included 758 women and exams (Fig. 2). The proportion of cancer was 50% (379/758). Younger women, 40 to 55 years of age, constituted 52% (393/758) of the entire dataset, 64% (243/379) of the healthy and 40% (150/379) of the diagnosed. Among 379 exams with cancer, there were 39 cases (10%) with in situ only, 161 cases (42%) with up to 15-mm invasive cancer, and 170 cases (45%) with more than 15-mm invasive cancer.

### Reader flagging rate

Reader outcomes are reported in Table 1. In the non-enriched population-wide original setting, there was a 34% (258/758) flagging rate by radiologists (without AI), while in the enriched study population, the reader study radiologists flagged 59% (444/758) of exams without AI and 46% (352/758) of exams with AI decision support. For standalone AI, pre-calibrated in a non-enriched setting, the flagging rate was 36% (352/758) of exams.

**Table 1:** Screening metrics for each reading regimen: original screening radiologists, study radiologists with and without AI, and standalone AI

	Read-and-consensus^a^				Read^b^
	Original screening	Reader study radiologists			Standalone AI
	Radiologists	Without AI	With AI assistance	Relative change	
Flagging rate^c^	34% (258/758)	59% (444/758)	46% (352/758)	− 21% (*p* < 0.001)	36% (276/758)
Positive predictive value	93% (241/258)	72% (318/444)	84% (295/352)	+ 17% (*p* < 0.001)	95% (261/276)
Sensitivity	64% (241/379)	84% (318/379)	78% (295/379)	− 7% (*p* = 0.017)	69% (261/379)
Specificity	96% (362/379)	67% (253/379)	85% (322/379)	+ 27% (*p* < 0.001)	96% (364/379)
Recall rate^d^	31% (238/758)	57% (431/758)	44% (337/758)	− 22% (*p* < 0.001)	N/A
Positive predictive value	97% (232/238)	71% (307/431)	84% (284/337)	+ 18% (*p* = 0.022)	N/A
Sensitivity	61% (232/379)	81% (307/379)	75% (284/379)	− 7% (*p* < 0.001)	N/A
Specificity	98% (373/379)	67% (255/379)	86% (326/379)	+ 28% (*p* < 0.001)	N/A

^a^For “Read-and-consensus” with AI assistance, image prompts and AI scores were available to the radiologists

^b^For “Read” based on standalone AI, the threshold for flagging was predefined in a prior study at 40 (range 0 to 100)

^c^First, in the individual read, “flagging” was done for any suspected abnormality

^d^Second, the consensus discussion was performed for all flagged cases and a “recall” or “no recall” decision was made

### Sensitivity and specificity after consensus discussion

**Fig. 3:**
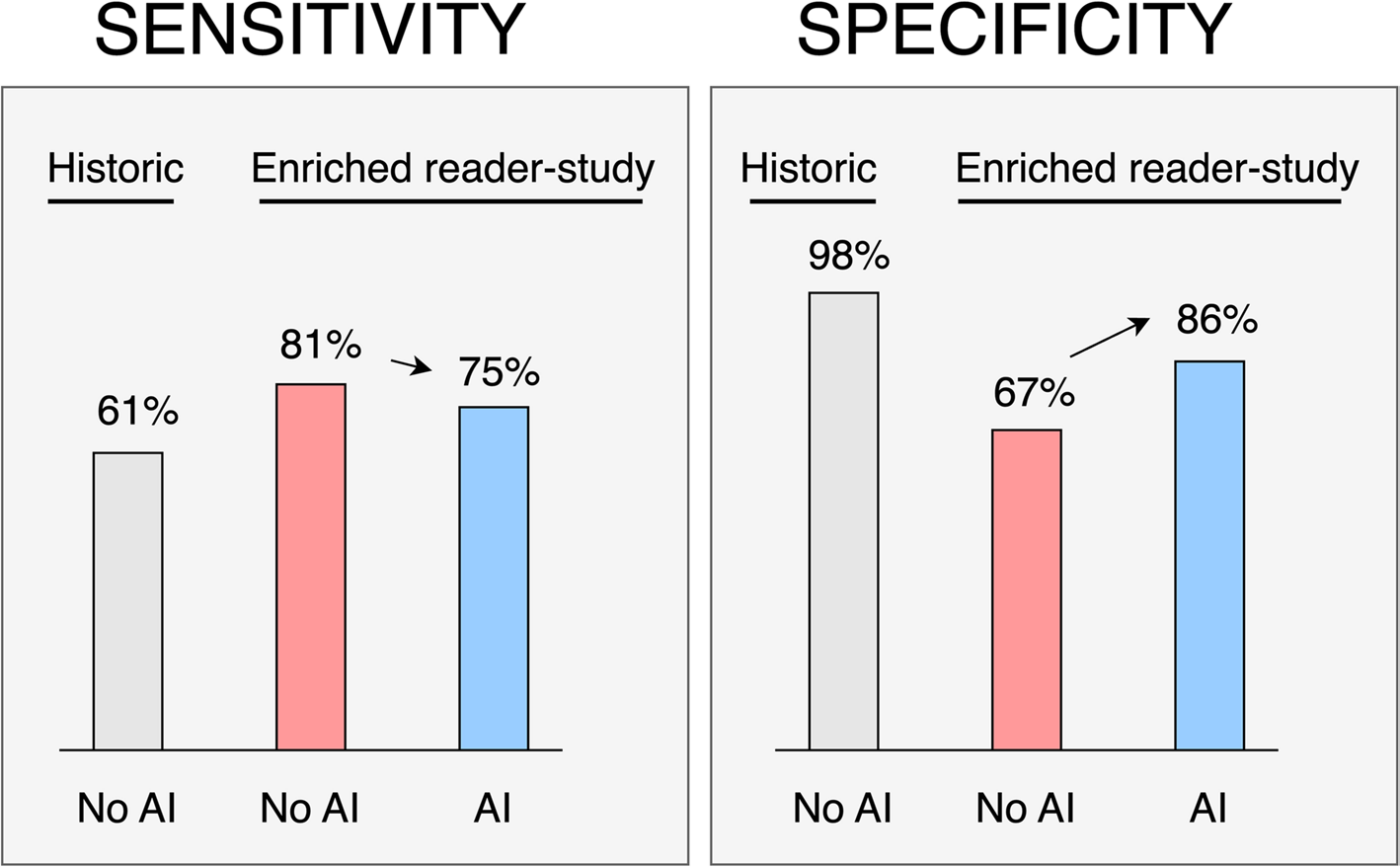
Results after double-reading including consensus discussion in screening mammography. The gray bars show the historic performance when the exam was assessed in population-wide screening. The red and blue bars show the performance, without (red) and with (blue) AI decision support, when the exams were assessed in a reader study with an enriched cohort with 50% cancer out of 758 exams. AI had been calibrated to perform similar to radiologists in population-wide screening

Compared to the original setting sensitivity of 61% (232/379), the reader study sensitivity was higher at 81% (307/379) without AI, which decreased to 75% (284/379) with AI decision support, a relative decrease of 7% (*p* < 0.001). Compared to the original setting specificity of 98% (373/379), the reader study specificity was lower at 67% (255/379) without AI, which increased to 86% (326/379) with AI decision support, a relative increase of 28% (*p* < 0.001). Figure 3 shows the shifts in sensitivity and specificity—between the original non-enriched screening setting and the highly cancer-enriched reader study setting with and without AI. When using AI, 39 of the cancers detected by study radiologists without AI were missed, and 16 cancers previously missed were detected with AI, resulting in a net decrease of 23 cancers detected when using AI decision support.

**Fig. 4:**
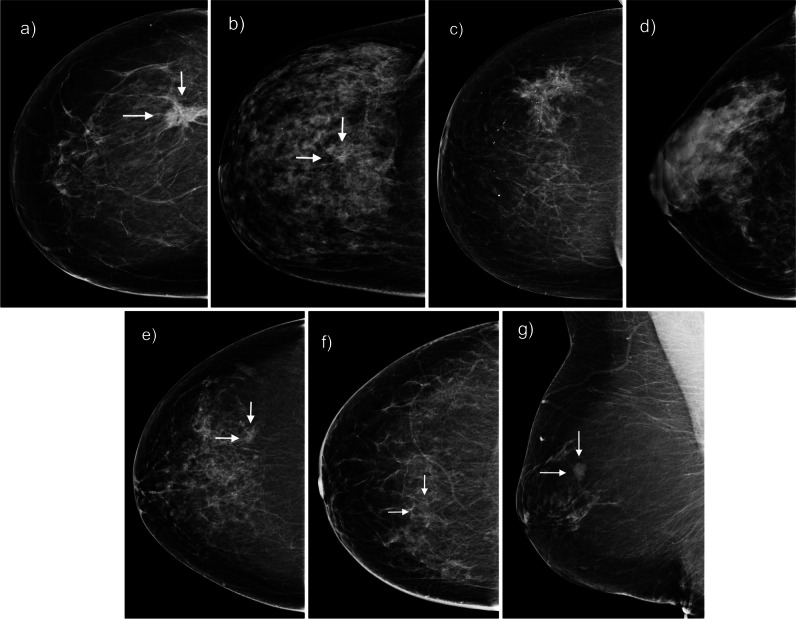
Example mammograms. **a** Cancer with AI score 99. **b** Cancer with AI score 40. **c** Healthy with AI score 40. **d** Healthy with AI score 0. **e** Cancer missed by AI but flagged by reader study radiologists. **f** Cancer missed by screening radiologists but flagged by AI and reader study radiologists. **g** Cancer detected by study radiologists without AI but missed with AI support

Figure 4 shows example mammograms. Panels a to d show example of mammograms with varying AI scores and healthy or diagnosed status. Panel e shows the mammogram of a cancer missed by AI but flagged by reader study radiologists. Panel f shows the mammogram of cancer missed by screening radiologists but flagged by AI. Finally, panel g shows cancer at first detected by study radiologists without AI but missed later when using AI support.

### Tendency to change assessment—radiologists, Bi-RADS, and image signs

In the reader study, Table 2 reports the proportion of study radiologists changing their assessment after reviewing AI information. The tendency to change assessment is further characterized by the Bi-RADS score assigned prior to AI and which lesion signs were present in the mammogram. Changing from negative to positive occurred twice for reader one and four times for reader two. Changing from positive to negative occurred 66 times for reader one and 102 times for reader two. AI decision support more often caused a change in radiologist read for Bi-RADS exams 3 (reader one changed their read in 30% (64/215) of these exams while reader two changed their read in 48% (97/201)) than for Bi-RADS 4 and 5 (five exams or less). Changing from positive to negative for Bi-RADS 3 was correct, i.e., the screened woman did not have cancer, in 61% (39/64) of cases for reader one and 70% (68/97) of cases for reader two. Changing from a positive to a negative read based on erroneous AI information was more common for exams with subtler signs of possible malignancy such as architectural distortion and asymmetrical density compared to more poignant signs such as microcalcifications. The higher Bi-RADS score and the higher number of different image signs of potential malignancy that were present, the less likely were the radiologists to change from positive to negative after reviewing AI information.

**Table 2:** Characteristics of exams where study radiologists changed assessment after reviewing AI information—from positive to negative, and from negative to positive

	Reader one^a^—changed assessments after reviewing AI information				Reader two^b^—changed assessments after reviewing AI information			
	Positive to negative	Truly negative	Negative to positive	Truly positive	Positive to negative	Truly negative	Negative to positive	Truly positive
Total	21% (66/321)	62% (41/66)	0.5% (2/437)	50% (1/2)	41% (102/251)	69% (70/102)	0.8% (4/507)	100% (4/4)
Bi-RADS								
3	30% (64/215)	61% (39/64)	1% (2/217)	50% (1/2)	48% (97/201)	70% (68/97)	0.5% (1/202)	100% (1/1)
4	3% (1/31)	100% (1/1)	0%	N/A	10% (5/50)	40% (2/5)	4% (2/52)	100% (2/2)
5	1% (1/75)	100% (1/1)	0%	N/A	0%	N/A	0.6% (1/154)	100% (1/1)
Type of image sign								
Calcification	8% (8/100)	38% (3/8)			8% (9/115)	22% (2/9)		
Mass	16% (38/244)	50% (19/38)			13% (35/274)	71% (25/35)		
Asymmetry	23% (17/75)	82% (14/17)			19% (17/91)	94% (16/17)		
Architectural distortion	29% (29/100)	79% (23/29)	*Insufficient data*		17% (17/102)	76% (13/17)	*Insufficient data*	
Skin thickening	9% (1/11)	0%			8% (1/12)	0%		
Nipple retraction	20% (2/10)	100% (2/2)			40% (6/15)	83% (5/6)		
Axillary lymph node	15% (3/20)	100% (3/3)			10% (2/21)	100% (2/2)		
Number of image signs								
1	25% (40/162)	58% (23/40)			24% (44/184)	64% (28/44)		
2	22% (23/105)	70% (16/23)	*Insufficient data*		17% (20/118)	80% (16/20)	*Insufficient data*	
3 or more	6% (4/58)	75% (3/4)			2% (1/64)	100% (1/1)		

^a^The “Reader one” position was shared between two study radiologists

^b^The “Reader two” position was assigned to a single study radiologist who was more experienced

Between the first assessments without AI, there was a 6-week wash-out period before the radiologists again reviewed the exams with AI assistance including image prompts and score

### Cancer characteristics

In Table 3, we report the characteristics of each cancer subgrouped by “recalled” and “not recalled” by each reading strategy. For the reading study, the number of invasive cancers larger than 15 mm that were detected was 134 and 128 without and with AI, respectively, while the number of invasive cancers 15 mm or smaller that were detected was 134 and 119 respectively. Thus, the erroneous change from positive to negative assessments was less frequent for larger than for smaller invasive cancers.

**Table 3:** Characteristics of cancers detected and not detected by each reading strategy

			Original screening		Reader study w/o AI		Reader study with AI		AI standalone	
		All	Recalled	Not recalled	Recalled	Not recalled	Recalled	Not recalled	Flagged	Not flagged
*n*		379	232	147	307	72	284	95	261	118
Dense area^a^	Mean cm^2^	36.7	33.9	44.1	35.5	41.8	35.5	40.1	35.3	39.7
Percent density^a^	Mean %	24.5	22.7	27.4	23.4	29.4	23.3	28.0	23.4	26.9
In situ only	% (*n*)	10 (39)	12 (29)	7 (10)	11 (35)	6 (4)	11 (32)	7 (7)	11 (29)	9 (10)
Invasive < = 15 mm	% (*n*)	42 (161)	44 (102)	40 (59)	44 (134)	38 (27)	42 (119)	44 (42)	41 (106)	47 (55)
Invasive > 15 mm	% (*n*)	45 (170)	42 (98)	49 (72)	44 (134)	50 (36)	45 (128)	44 (42)	48 (124)	39 (46)
Missing information	% (*n*)	2 (9)	1 (3)	4 (6)	1 (4)	7 (5)	2 (5)	4 (4)	1 (2)	6 (7)
Lymph node negative	% (*n*)	66 (251)	71 (164)	59 (87)	68 (208)	60 (43)	69 (196)	58 (55)	69 (179)	61 (72)
Lymph node positive	% (*n*)	27 (103)	22 (51)	35 (52)	25 (77)	36 (26)	24 (68)	37 (35)	25 (66)	31 (37)
Missing information	% (*n*)	7 (25)	7 (17)	5 (8)	7 (22)	4 (3)	7 (20)	5 (5)	6 (16)	8 (9)

^a^Dense area and percent density were calculated by the Libra software package from University of Pennsylvania (https://www.med.upenn.edu/cbica/sbia/libra.html)

### Reading time

The mean time for double-reading an exam without AI was 21 s (median 19; p25, 16; p75, 24), and with AI, it was 13 s (median 13; p25, 11; p75, 15), a relative change of minus 38%.

## Discussion

In our strongly cancer-enriched study population, AI decision support resulted in 7% decrease in sensitivity, 28% increase in specificity, and 38% decrease in radiologist time. Compared to the study radiologists without AI decision support, the original screening assessments had a markedly lower sensitivity and a markedly higher specificity.

Compared to a Bi-RADS minimum acceptable sensitivity of 75%, defined by a 1-year follow-up time, the measured average sensitivity is lower in Sweden due to having a 2-year follow-up time during which further interval cancer is collected increasing the denominator.

The reading times in our study differ from a previous study reporting reading times of 63 s without AI and 72 s with AI [12]. The much shorter times in our study might be related to that we measured only the time from when images were displayed until the flag or no-flag decision. In the reading time, we did not include the further characterization of image findings. In addition, our readers were not shown previous images so all four images were shown at once in a single layout. We are not aware of any difference in study methodology compared to the previous study that would explain our observation that the reading time decreased when using AI while the time in the previous study increased. A speculation is that it may be related to how and when the AI information was displayed.

The markedly higher sensitivity of reader study radiologists compared to original assessments may seem odd. We interpret this as the result of study radiologists gradually realizing there is a high proportion of cancer in the population, and subconsciously shifting their operating point as a response to a shift in the cost–benefit ratio of flagging an exam. This is an important consideration when implementing AI as a triaging tool for screening mammography. Several studies have focused on using AI to place exams into ten categories according to the expected prevalence of cancer [6, 7, 13]. They could demonstrate that the top category contained most of the screen-detected and many of the interval cancers. However, those studies are limited by extracting the original screen-read interpretation, not fully taking into account the bias of radiologists knowing the category of the exam. In line with our results, in one reader study, radiologists reading the high-prevalence list seemed to shift their operating point towards higher sensitivity and lower specificity, increasing cancer yield at the cost of some decrease in specificity [7]. Accordingly, radiologists reading the low-prevalence category might be expected to shift their operating point towards lower sensitivity and higher specificity. The shift in study radiologist operating point caused by concurrent AI decision support demonstrates automation bias which is in line with the results of two recent publications [8, 9]. In our study, radiologists changed their assessment from positive to negative in 21 to 41% of the exams even though this was incorrect in 31 to 38% of cases. This is in line with the findings of the study by Dratsch et al, in which the readers incorrectly followed the AI information in 20 to 45% of the cases. In the study by Rezazade Mehrizi et al, radiologists missed 16% proportion of cancers due to erroneous AI information. In our study, we observed that the likelihood that readers would miss a cancer due to erroneous AI information was related to the perceived level of evidence for malignancy. When prior to reviewing AI information they assigned the exam a higher Bi-RADS score, more poignant and higher number of image signs, they less often changed from a positive to negative read.

Conceptually, there seem to be two types of automation bias: first, a nudging of the radiologist operating point towards the operating point of AI with false-negative and false-positive mistakes changing in opposite directions; and second, an increase in any sort of mistake due to overreliance on erroneous AI suggestions. Automation bias of the first type, deliberately exploited, might be beneficial in terms of improving the benefit-to-harms ratio of screening, detecting more cancer in high-risk groups and avoiding false-positives in low-risk groups. The phenomenon of radiologist being nudged towards the operating point of the AI assistant would help to reduce interreader variability and ensure a more equal screening operating point for all participants. Automation bias of the second type, however, is better avoided. Four different countermeasures were suggested in a recent commentary [14]. In one mentioned study, the researchers chose to have AI running in the background and only interfering when the radiologists were at risk of making an obvious false-negative or false-positive mistake [15].

A strength of our study was the simulation of the entire radiologist workflow—both the initial read and the following consensus discussion. Another strength is that we used an AI system that has shown a highly accurate performance in prior retrospective studies and a recent prospective study [4, 5, 16]. A limitation of our study is that the enrichment ratio was extremely high which may not often be the case even in high-risk triaging implementations. A weakness of our study is that it was not the same radiologists that performed the original screening read as were involved in the reader study. However, they all operate in the same type of population-based screening environment.

In conclusion, radiologists’ shift in sensitivity and specificity was affected by the cancer prevalence in the reading list, by the operating point of the AI, and by the type and number of image signs. When AI is implemented for triaging, the sensitivity and specificity of radiologists can be expected to change depending on the perceived cancer prevalence in the exams triaged to high risk and low risk respectively. When AI is implemented as a concurrent assistant, the radiologists can be expected to change their operating point towards the one decided for the AI system. These interaction effects may not be possible to estimate beforehand, calling for careful monitoring of radiologist performance, overall and individually, in real-world implementations of AI for screening mammography.

## Abbreviations

AI: Artificial intelligence
Bi-RADS: Breast Imaging Reporting and Data System
CAD: Computer-aided detection
CSAW-CC: Cohort of screen-age women—case–control

## Declarations

### Guarantor

The scientific guarantor of this publication is Fredrik Strand, Karolinska Institutet.

### Statistics and biometry

One of the authors (Fredrik Strand) has significant statistical expertise.

No complex statistical methods were necessary for this paper.

### Study subjects or cohorts overlap

Some study subjects or cohorts have been previously reported in doi: 10.1001/jamaoncol.2020.3321 and doi.org/10.1016/S2589-7500(20)30185-0, doi.org/10.1148/radiol.2019190872.

### Methodology

•retrospective

•case-control study

•performed at one institution

## References

[1] Lång K, Hofvind S, Rodríguez-Ruiz A, Andersson I (2021) Can artificial intelligence reduce the interval cancer rate in mammography screening? Eur Radiol 31:5940–5947 10.1007/s00330-021-07686-3 PMC8270858 33486604

[2] Dembrower K, Salim M, Eklund M, Lindholm P, Strand F (2023) Implications for downstream workload based on calibrating an artificial intelligence detection algorithm by standalone-reader or combined-reader sensitivity matching. J Med Imaging (Bellingham) 10(S2):S22405–S22405 10.1117/1.JMI.10.S2.S22405 PMC10075995 37035276

[3] Yoon JH, Strand F, Baltzer PAT et al(2023) Standalone AI for breast cancer detection at screening digital mammography and digital breast tomosynthesis: a systematic review and meta-analysis. Radiology 307(5):e222639. 10.1148/radiol.222639 10.1148/radiol.222639 PMC10315526 37219445

[4] Salim M, Wåhlin E, Dembrower K et al (2020) External evaluation of 3 commercial artificial intelligence algorithms for independent assessment of screening mammograms. JAMA Oncol. 6(10):1581–1588. 10.1001/jamaoncol.2020.3321 10.1001/jamaoncol.2020.3321 PMC7453345 32852536

[5] Dembrower K, Wåhlin E, Liu Y (2020) Effect of artificial intelligence-based triaging of breast cancer screening mammograms on cancer detection and radiologist workload: a retrospective simulation study. Lancet Digit Health 2(9):e468–e474 10.1016/S2589-7500(20)30185-0 33328114

[6] Lauritzen AD, Rodríguez-Ruiz A, von Euler-Chelpin MC et al (2022) An artificial intelligence–based mammography screening protocol for breast cancer: outcome and radiologist workload. Radiology 304(1):41–49 10.1148/radiol.210948 35438561

[7] Rodríguez-Ruiz A, Krupinski E, Mordang JJ et al (2019) Detection of breast cancer with mammography: effect of an artificial intelligence support system. Radiology 290(2):305–314 10.1148/radiol.2018181371 30457482

[8] Dratsch T, Chen X, Mehrizi MR et al (2023) Automation bias in mammography: the impact of artificial intelligence BI-RADS suggestions on reader performance. Radiology 307(4):e222176 10.1148/radiol.222176 37129490

[9] Rezazade Mehrizi MH, Mol F, Peter M et al (2023) The impact of AI suggestions on radiologists’ decisions: a pilot study of explainability and attitudinal priming interventions in mammography examination. Sci Rep 13(1):9230 10.1038/s41598-023-36435-3 PMC10247804 37286665

[10] Galati F, Moffa G, Pediconi F (2022). Breast imaging: beyond the detection. Eur J Radiol.

[11] Dembrower K, Lindholm P, Strand F (2020). A multi-million mammography image dataset and population-based screening cohort for the training and evaluation of deep neural networks—the Cohort of Screen-Aged Women (CSAW). J Digit Imaging.

[12] Pacilè S, Lopez J, Chone P et al (2020) Improving breast cancer detection accuracy of mammography with the concurrent use of an artificial intelligence tool. Radiology Artif Intel 2(6):e190208 10.1148/ryai.2020190208 PMC8082372 33937844

[13] Larsen M, Aglen CF, Lee CI et al (2022) Artificial intelligence evaluation of 122 969 mammography examinations from a population-based screening program. Radiology 303(3):502–511 10.1148/radiol.212381 PMC9131175 35348377

[14] Baltzer PAT (2023). Automation bias in breast AI. Radiology.

[15] Leibig C, Brehmer M, Bunk S, Byng D, Pinker K, Umutlu L (2022) Combining the strengths of radiologists and AI for breast cancer screening: a retrospective analysis. Lancet Digit Health 4(7):e507–e519 10.1016/S2589-7500(22)00070-X PMC9839981 35750400

[16] Dembrower K, Crippa A, Colón E, Eklund M, Strand F; ScreenTrustCAD Trial Consortium (2023) Artificial intelligence for breast cancer detection in screening mammography in Sweden: a prospective, population-based, paired-reader, non-inferiority study. Lancet Digit Health 5(10):e703-e711. 10.1016/S2589-7500(23)00153-X 10.1016/S2589-7500(23)00153-X 37690911

